# 

**Published:** 1885-06

**Authors:** 


### CONTENTS.

### PROCEEDINGS.

Southern Dental Association .......... 265

SALMAGUNDI .......... 327

### EDITORIAL.

Indiana State Dental Association .......... 334

Southern Dental Association .......... 335

Dissolution .......... 335

Obituary .......... 335

